# Noninvasive electrocardiographic imaging of dynamic atrioventricular delay programming in a patient with left bundle branch block

**DOI:** 10.1016/j.hrcr.2021.09.009

**Published:** 2021-09-29

**Authors:** Peter H. Waddingham, Jan Mangual, Michele Orini, Nima Badie, Luke McSpadden, Pier D. Lambiase, Anthony W.C. Chow

**Affiliations:** ∗ Barts Heart Centre, St. Bartholomew’s Hospital, West Smithfield, London, United Kingdom; † Abbott, Sylmar, California; ‡ Institute of Cardiovascular Science, University College London, London, United Kingdom; § William Harvey Research Institute, Charterhouse Square, Queen Mary University of London, London, United Kingdom

**Keywords:** CRT optimization, SyncAV, Electrocardiography imaging, Atrioventricular delay, MultiPoint Pacing

## Introduction

The response to cardiac resynchronization therapy (CRT) is determined by various factors, including left ventricular (LV) lead location, atrioventricular (AV) delay, and inter-/intraventricular delays. Advances in quadripolar lead technology and device algorithms have improved patient response, yet selection of optimal settings remains challenging. Studies have shown acute improvement in electrical synchrony with manual AV optimization by fusion optimized intervals^1^^,^^2^; automated device algorithms, for example AdaptivCRT (Medtronic, Minneapolis, MN),^3^ SmartDelay (Boston Scientific, Marlborough, MA),^4^ and SyncAV^TM^ (Abbott, Sylmar, CA)^5^; and pacing from multiple LV lead electrodes with MultiPoint Pacing (MPP).^6^^,^^7^ The aim of this clinical case report was to evaluate the acute benefits of SyncAV Plus in the new-generation, Bluetooth-enabled Gallant^TM^ CRT device (Abbott, Sylmar, CA). SyncAV Plus continually programs the paced AV delay shorter than the intrinsic PR interval by a programmable offset (% of PR duration) to synchronize intrinsic and ventricular paced activation wavefronts. Twelve-lead electrocardiogram (ECG) and noninvasive electrocardiographic imaging (ECGi) epicardial mapping analyses were performed to characterize the impact of SyncAV Plus on electrical synchrony during a range of CRT programming strategies, including biventricular (BiV) pacing, MPP, LV-only pacing, and LV-only pacing with MPP.

## Case report

**Figure 1:**
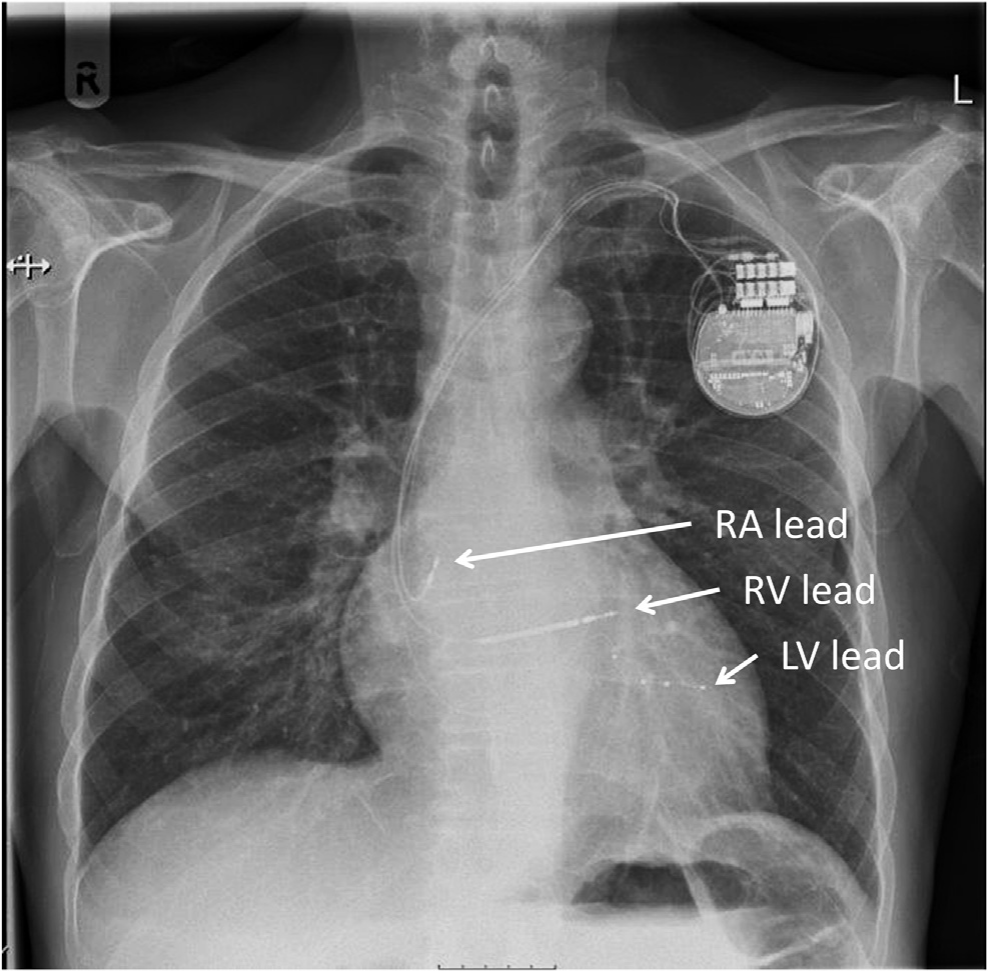
Chest radiograph displaying device and lead positions. This posteroanterior projection shows the final positions of the device and leads. Left ventricular (LV) lead was placed at the basal-mid posterolateral branch of the coronary sinus, right ventricular (RV) lead at the RV apex, and right atrial (RA) lead in the RA appendage.

A 75-year-old male patient was referred for elective primary prevention CRT-D device implantation. Past medical history included hypertension, type 2 diabetes mellitus, and ischemic cardiomyopathy with left bundle branch block (LBBB). Echocardiography showed severe LV systolic impairment (LV ejection fraction 30%) with moderate mitral regurgitation. Twelve-lead ECG showed wide QRS (178 ms) and PR interval 159 ms, and the patient was symptomatic of heart failure, NYHA functional class II, despite optimal medical therapy. An Abbott Gallant CRT-D was implanted, with a quadripolar LV lead (Quartet 1458Q) placed in a lateral branch of the coronary sinus and the right ventricular (RV) lead in the RV septum. Figure 1 illustrates the final postprocedure lead positions. At the 4 LV electrodes (D1/M2/M3/P4), Q-LV times were 166/143/143/166 ms and device-measured RV-sensed to LV-sensed conduction times were 140/154/154/157 ms.

The following pacing configurations were evaluated: intrinsic conduction (no RV or LV pacing), BiV pacing (BiV = RV + LV1), MPP (MPP = RV + LV1 + LV2), LV-only single-site pacing (LVSS = LV1 only), and LV-only MPP (LVMPP = LV1 + LV2). LV pacing was delivered for BiV and LVSS using the latest activating electrode as the cathode (P4), and for MPP and LVMPP using the electrodes with the widest anatomical spacing as the cathodes (D1 and P4). All configurations used the minimum programmable RV-LV1 and LV1-LV2 delays. For each pacing configuration, SyncAV Plus was activated with the offset (5%–30% of the right atrial [RA]-RV sensed interval, ie, PR interval) resulting in the narrowest QRSd. The optimal SyncAV Plus offsets identified were 10% for BiV, 30% for MPP, 20% for LVSS, and 40% for LVMPP.

**Figure 2:**
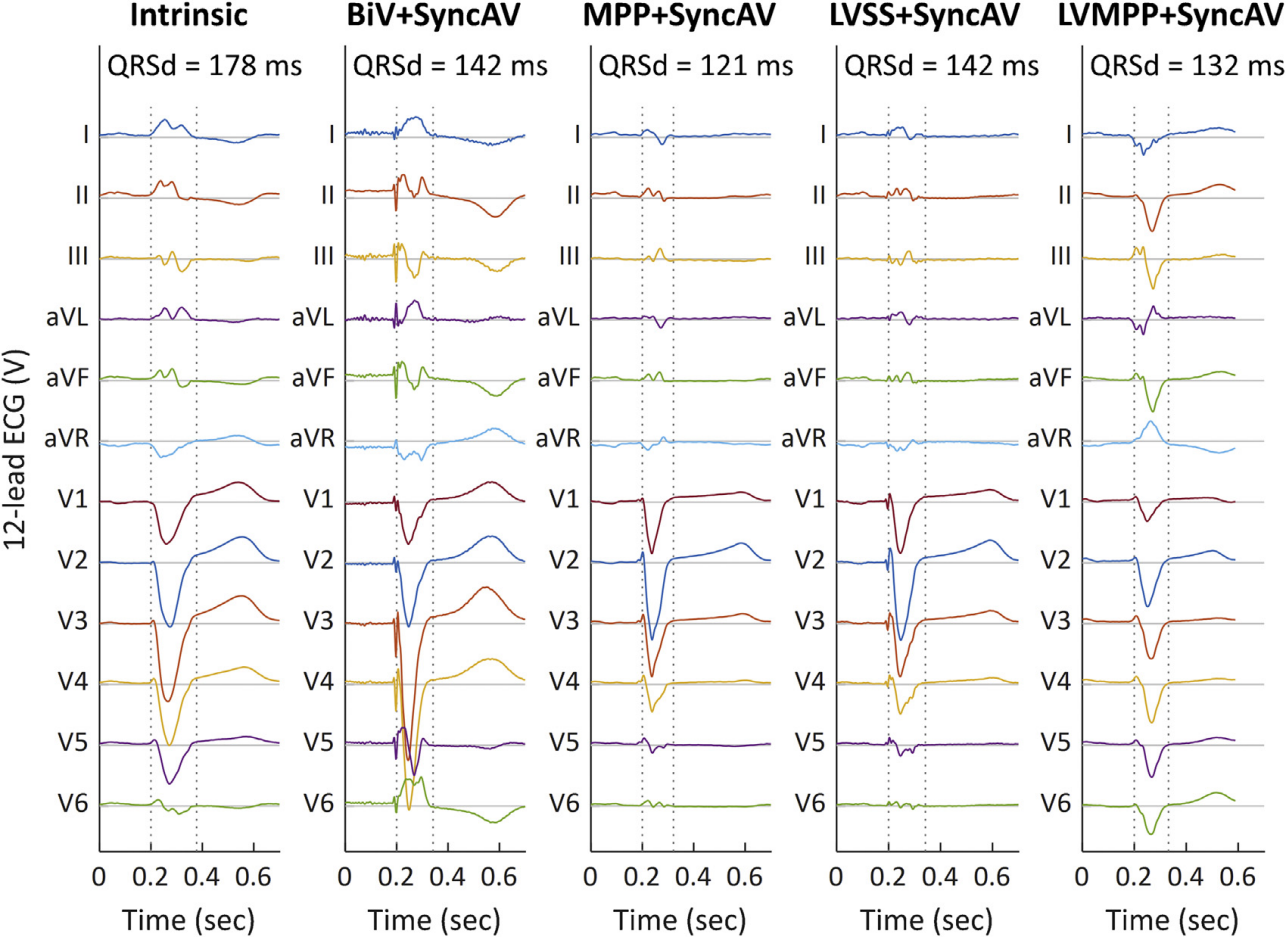
Twelve-lead surface electrocardiogram (ECG). The 12-lead surface ECG ensemble signals averaged over 3 successive beats are displayed for intrinsic conduction, biventricular (BiV) + SyncAV, Multi-Point Pacing (MPP) + SyncAV, left ventricle–only single-site pacing (LVSS) + SyncAV, and LV-only MPP (LVMPP) + SyncAV (left to right). QRS durations (QRSd) are displayed, with QRS start and end times shown as dashed vertical lines.

The QRSd for each configuration was measured by manually annotating 12-lead surface ECG, measuring from first deflection of the QRS complex (excluding the pacing spike) to return to baseline across all leads. Table 1 and Figure 2 display the QRSd values and change with respect to intrinsic rhythm for each pacing strategy. Relative to the QRSd during intrinsic conduction (178 ms), BiV+SyncAV reduced QRSd by 20.2% to 142 ms, and MPP+SyncAV reduced QRSd by 32.0% to 121 ms. Taking advantage of intact right bundle branch activation, LVSS+SyncAV reduced QRSd by 20.2% to 142 ms, and LVMPP+SyncAV reduced QRSd by 25.8% to 132 ms.

**Table 1:** Summary of 12-lead electrocardiogram and electrocardiographic imaging mapping data

ECG & ECGi data	Intrinsic	BiV + SyncAV	MPP + SyncAV	LVSS + SyncAV	LVMPP + SyncAV
QRSd, ms (% reduction vs intrinsic)	178	142 (20%)	121 (32%)	142 (20%)	132 (26%)
LVAT, ms (% reduction vs intrinsic)	142	124 (13%)	94 (34%)	128 (10%)	119 (16%)
LVED, ms (% change vs intrinsic)	0.0232	0.0227 (-2.2%)	0.0153 (-34.1%)	0.0270 (+16.4%)	0.0171 (-26.3%)
VEU, ms (% change vs intrinsic)	58	40 (31%)	0.30 (99%)	32 (44%)	5 (92%)

BiV = biventricular; ECG = electrocardiogram; ECGi = electrocardiographic imaging LVAT = left ventricular activation time; LVED = left ventricular electrical dispersion; LVMPP = left ventricle–only Multi-Point Pacing; LVSS = left ventricle–only single-site pacing; MPP = MultiPoint Pacing; VEU = ventricular electrical uncoupling.

Noninvasive epicardial ECGi was performed using a high-resolution mapping system (CardioInsight; Medtronic, Minneapolis, MN), as previously described.^8^^,^^9^ A 252-electrode ECGi vest was fitted to the patient’s torso, followed by a low-dose noncontrast thoracic computed tomography to acquire electrode-cardiac geometry. The total LV activation times (LVAT, latest − earliest activation time on the left ventricle), electrical dispersion (LVED coefficient of variance = standard deviation of LV activation times / mean LVAT) and ventricular electrical uncoupling (VEU = mean LVAT – mean RV activation time) were averaged across 3 independent QRS complexes for intrinsic conduction and each pacing configuration.

**Figure 3:**
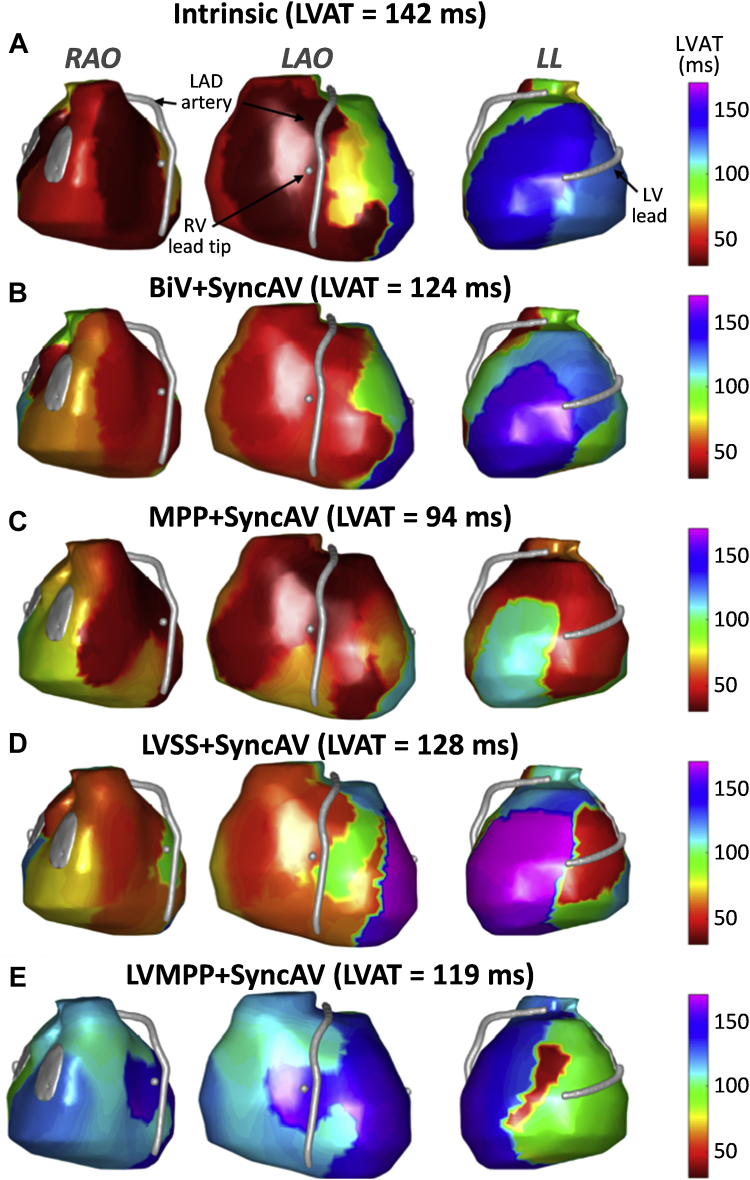
Electrocardiographic imaging activation maps. Epicardial ventricular surfaces of both ventricles are displayed in 3 views: from left to right, right anterior oblique (RAO), left anterior oblique (LAO), and left lateral (LL). The left anterior descending (LAD) artery, right ventricle (RV) lead tip, and left ventricle (LV) lead are depicted as silver surfaces. Color maps are projected onto the surface for activation time (AT, ms), ranging from early (red/orange) to late (blue/purple). Intrinsic conduction activation maps show the earliest-activating regions in the anterior RV (inclusive of septal activation) and RV free wall in keeping with intact right bundle conduction. The latest-activating LV segment is basal lateral, and a U-shaped line of activation discontinuity is present from the basal anterior-anterolateral LV spreading apically to the basal posterior LV. The impact of each pacing modality on activation pattern is displayed: **A:** intrinsic conduction; **B:** biventricular (BiV) + SyncAV; **C:** MultiPoint Pacing (MPP) + SyncAV; **D:** LV-only single-site pacing (LVSS) + SyncAV; **E:** LV-only MPP (LVMPP) + SyncAV.

Example ventricular activation maps are illustrated in Figure 3. Intrinsic conduction activation maps show earliest activating regions (red/orange) in the anterior right ventricle (inclusive of septal activation) and RV free wall, in keeping with intact right bundle conduction. The latest-activating LV segment (blue/purple) is basal-lateral, and the LV lead location is concordant with this segment.

There is a U-shaped line of activation discontinuity with a high gradient of activation time, evident by isochronal crowding present from the anterior-anterolateral LV around to the basal posterior LV. The wavefront of LV activation did not spread from the anterior to lateral wall but traveled inferoapically before traveling across the inferior LV wall to finally activate the basal-lateral segment. This is in keeping with patterns of activation previously described in LBBB by 3-D contact and noncontact mapping studies.^10^^,^^11^ The line of activation discontinuity was partially altered across all LV pacing modalities, with the latest activating segment shifting anteriorly and LV activating earlier and appearing more homogenous. In this case, the activation discontinuity may have had a functional element and was at least partially modifiable. MPP and LV-only MPP maps show a greater degree of activation pattern modification vs BiV and LV-only maps with the line of activation discontinuity reducing in extent.

Quantitative assessment of LV activation characteristics demonstrated that LVAT during intrinsic conduction (142 ms) was reduced by all pacing configurations: BiV+SyncAV (124 ms, 13% reduction with respect to intrinsic), MPP+SyncAV (94 ms, 34% reduction), LVSS+SyncAV (128 ms, 10% reduction), and LVMPP+SyncAV (119 ms, 16% reduction). Table 1 displays the LVAT data and change with respect to intrinsic rhythm. The LVED during intrinsic conduction (0.0232) was reduced by BiV+SyncAV (0.0227, 2.2% reduction), MPP+SyncAV (0.0153, 34.1% reduction), and LVMPP+SyncAV (0.0171, 26.3% reduction) but increased with LVSS+SyncAV (0.0270, 16.4% increase). VEU was also reduced with all pacing configurations with respect to intrinsic conduction (58 ms): BiV+SyncAV (40 ms, 31% reduction with respect to intrinsic), MPP+SyncAV (0.30 ms, 99% reduction), LVSS+SyncAV (32 ms, 44% reduction), and LVMPP+SyncAV (5 ms, 92% reduction).

The patient’s postprocedure recovery was uneventful, without any procedure-related complications. The device was programmed to MPP with 30% SyncAV offset as part of an ongoing clinical study (NCT03567096). At follow-up, the patient was well, describing an improvement in exertional capacity; the device and lead parameters were stable with 99% BiV pacing.

## Discussion

This is the first case report to demonstrate the effect of SyncAV Plus on synchronizing LV activation, as evaluated by ECGi in a patient with LBBB and intact AV conduction. The Gallant CRT device used in this patient provides the unique advantage of customizable percentage-based SyncAV Plus offsets, potentially providing more consistent dynamic AV interval adjustment during CRT pacing. Enhancements in acute electrical synchrony have been previously shown with SyncAV,^5^^,^^7^ via 12-lead ECG QRSd reduction. Enabling and optimizing SyncAV Plus improved QRSd, LVAT, and VEU in all pacing configurations evaluated. LVED improved in all pacing configurations except LVSS+SyncAV. Activation maps showed that LV activation patterns were significantly modified, resulting in more homogenous LV activation and reduced activation discontinuity, with greater impact demonstrated by MPP and LVMPP. This patient received incremental improvement in electrical synchrony even with LV-only pacing (single-site and MultiPoint), highlighting the potential benefit of SyncAV Plus and MPP for patients with intact right bundle conduction, if battery longevity is a concern.

The case presented here involves a single patient with ischemic cardiomyopathy undergoing ECGi. These results, although of interest, must be evaluated in a larger cohort with varying underlying substrates to better understand the mechanism of AV optimization with SyncAV.

## Conclusion

The acute benefits of SyncAV Plus on QRS duration and LV activation time were demonstrated across all evaluated pacing configurations. Incorporating RV pacing with MPP demonstrated incrementally superior QRS narrowing in this case, as compared to LV-only pacing with and without MPP.

**Key Teaching Points**

• SyncAV Plus (Abbott, Sylmar, CA) is a customizable cardiac resynchronization therapy device algorithm that automatically adjusts the atrioventricular (AV) delay, to synchronize intrinsic and paced conduction.
• SyncAV Plus may provide potential benefits to electrical synchrony across a range of pacing strategies, as illustrated in this case of a patient with intact AV conduction, left bundle branch block, and nonischemic cardiomyopathy. Strategies assessed included biventricular, left ventricular (LV)-only, biventricular MultiPoint Pacing (MPP), and LV-only MPP.
• Electrical synchrony was assessed by QRSd from 12-lead electrocardiogram and electrocardiographic imaging (ECGi) activation mapping. The acute benefits of SyncAV Plus on QRSd and LV activation time were demonstrated across all evaluated pacing configurations.
• Incorporating right ventricular pacing with MPP demonstrated incrementally superior electrical synchrony vs LV-only pacing with or without MPP.
• Noninvasive ECGi mapping provided detailed qualitative and quantitative data describing the impact of differing pacing strategies with SyncAV Plus.
